# Majority Voting-Based MAC Protocol for Exploiting Link-Layer Diversity in Wireless Networks †

**DOI:** 10.3390/s21082706

**Published:** 2021-04-12

**Authors:** Jaehyoung Park, Yonggang Kim, Gyungmin Kim, Hyuk Lim

**Affiliations:** 1 School of Electrical Engineering and Computer Science, Gwangju Institute of Science and Technology (GIST), 123, Cheomdangwagi-ro, Buk-gu, Gwangju 61005, Korea; jaehyoungpark@gist.ac.kr (J.P.); gyungminkim@gist.ac.kr (G.K.); 2 Samsung Electronics, Suwon 16677, Korea; Yonggang.kim@samsung.com; 3 Artificial Intelligence Graduate School, Gwangju Institute of Science and Technology, Gwangju 61005, Korea

**Keywords:** centralized WLAN, link-layer diversity combining, MAC protocol

## Abstract

In wireless local area networks (WLANs), the effect of interference signals between neighboring nodes increases as the number of wireless nodes using limited radio frequency resources in a limited space increases, which can significantly degrade the reliability of data transmission. In high-density WLANs, there can be several neighboring access points (APs) that can receive uplink transmission from a station. In conventional medium access control (MAC) protocols, uplink data frames containing errors or transmitted from a non-associated station are discarded at APs. Alternatively, we propose a MAC protocol using redundant wireless links between neighboring APs and the non-associated stations. In the proposed MAC protocol, we consider a centralized WLAN with a control node that performs error corrections of erroneous uplink data frames via a majority voting algorithm-based link-layer diversity scheme using uplink data received from multiple APs to increase the reliability of data transmission. In addition, we propose an adaptive carrier sensing ranging mechanism to improve the uplink network throughput in the proposed centralized WLAN system. Further, we conduct simulation studies and software-defined radio-based experiments to evaluate the performance of the proposed MAC protocol in various WLAN scenarios.

## 1. Introduction

In recent decades, remarkable advances in wireless network technology have made wireless services faster and more convenient, thereby increasing wireless local area network (WLAN) usage. Consequently, high-density WLAN (HD-WLAN) environments have increased significantly. However, in HD-WLAN scenarios where many access points (APs) use limited radio frequency resources, it is still difficult to support the user’s quality of service because inter-node interference in the HD-WLAN system is very high. The IEEE 802.11 standard, which is widely used in WLAN technology, adopts a distributed coordination function (DCF) based on a carrier sensing multiple access with collision avoidance (CSMA/CA) as a medium access control (MAC) protocol to support distributed operation of WLAN nodes. In the CSMA/CA protocol, the node checks the channel state before data transmission and then transmits a data frame, thereby mitigating performance degradation owing to frame collision or interference. However, in HD-WLAN environments, the use of the CSMA/CA protocol drastically reduces the average channel occupancy time of a WLAN node because many neighboring nodes in the transmission range of the node cannot access the channel while it is transmitting. This drastically degrades the wireless network performance [1].

In the case that a station transmits an uplink data frame in an HD-WLAN environment, the probability that multiple APs exist within the transmission range of the station is high, and the probability that multiple APs receive the data frame from the station is also high. In the existing MAC protocols for WLAN systems, if the destination MAC address of a data frame does not match the MAC address of an AP, or if the frame does not pass the cyclic redundancy check (CRC), the AP discards the received data frame. That is, only the associated AP receives the station’s uplink data frame if the frame passes through the CRC. Several diversity combining schemes have been proposed to increase the reliability of uplink data transmission by exploiting wireless channel diversity between a station and non-associated APs [2,3]. However, the existing proposed techniques focused on reducing the bit error rate (BER) using the diversity combining, but most of them incur high overhead for diversity combining at bit/symbol level. For example, the block-based bit-level diversity combing scheme in [2] has exponentially increasing computational complexity with respect to data length, and the symbol-level diversity combing scheme in [3] has a very large communication overhead for diversity combing using the symbols. Furthermore, they did not have compatibility with the existing WLAN MAC protocols because of the high computation/communication overhead. Therefore, it is highly required to develop a diversity combining scheme with low overhead in terms of computation and communication delays while guaranteeing compatibility with the existing WLAN nodes.

In this paper, we propose a diversity combining scheme that exploits the wireless channels between stations and APs to improve the performance of HD-WLANs by extending the paper in [4]. The proposed scheme adopts a signal-to-interference-plus-noise ratio (SINR)-based majority voting algorithm (MVA) with high reliability and efficiency. In high-density wireless networks exposed to high levels of interference, exploiting all connected AP information may not always be beneficial to the performance. Our proposed scheme utilizes SINR information from each AP to determine an AP set that maximizes the performance of the diversity combining by estimating the expected performance prior to performing the combining. Then, the MVA is performed for the frames relayed from the selected APs. When the number of APs is even and there exists an ambiguity of the combining result by MVA, the result of APs with high SINR is adopted. The proposed scheme has low computational complexity because each AP does not perform CRC for the frames to be relayed and the control node (CN) performs a simple MVA to the frame from a subset of available APs. Furthermore, as the proposed scheme can increase robustness against interference signals from neighboring nodes, nodes in the wireless network can use larger carrier sensing range to enable more nodes to transmit data simultaneously. A number of researches have been conducted to find optimal PCS ranges in various wireless networks [5] because network throughput and transmission reliability are significantly affected by the PCS range. However, research on the PCS mechanism in the WLAN environment where the diversity combining technique is applied is insufficient. We propose an adaptive carrier sensing (ACS) scheme that can increase network throughput in the systems to which link-layer diversity combining is applied. The main contributions of this work are summarized as follows.

- We propose a diversity combining scheme using an SINR-based MVA for high reliability and efficiency in HD-WLANs. In the proposed system, the AP selection algorithm determines an AP set that maximizes the performance of diversity combining, and the MVA-based diversity combining can efficiently achieve a reliable reconstruction of data frames from erroneous frames from the AP set. The proposed MAC protocol is also designed to guarantee compatibility with existing WLAN nodes by satisfying delay requirements with low computation and communication overhead.
- We propose an ACS mechanism to find a carrier sensing range that can improve the network throughput when the diversity combining scheme is applied in various HD-WLAN environments.
- To evaluate the performance of the proposed schemes, we perform simulation studies and software-defined radio (SDR)-based experiments in a variety of scenarios.

The rest of this paper is organized as follows. Section 2 presents the related works to analyze and improve the performance of high-density wireless networks. Section 3 describes the system model for diversity combining in HD-WLANs. In Section 4, we propose a MAC protocol for a centralized WLAN system that supports diversity combining and an ACS mechanism to improve the uplink throughput in the proposed system where diversity combining is applied. Section 5 presents the proposed AP selection algorithm and diversity combining scheme using an SINR-based MVA, and Section 6 provides computational and communication overhead analysis. Section 7 presents simulation and SDR-based experiment results to verify the performance of the proposed link-layer diversity combining and ACS schemes. Finally, Section 8 summarizes our study and concludes this paper.

## 2. Related Work

As the number of wireless nodes in use continues to increase due to the popularity of IoT and mobile devices, researches have been actively conducted to achieve high channel efficiency and reliability of dense wireless networks with a number of nodes. In [1], Bellalta derived a numerical model to approximate the throughput of high-density wireless networks by considering interactions between wireless networks and demonstrated the negative effect of frame collision using a continuous-time Markov chain in the HD-WLANs. In order to mitigate the network performance degradation caused by high interference levels and poor channel use in high-density wireless networks, many studies have been conducted from the viewpoint of the physical layer [6,7] and the link layer [8,9,10,11,12]. A dynamic channel bonding based on a carrier range was proposed in [6], and a PCS threshold selection scheme based on CSMA/CA was proposed to increase the efficiency of resource utilization in high-density wireless networks in [7]. Gurewitz et al. [8] proposed a MAC protocol that avoids collision by using a polling technique after the receiver receives a short signal containing the transmission intention in the wireless sensor network. In [9], the channel collision probability was reduced by optimizing channel reliability using the accumulated wireless channel information obtained by reinforcement learning. In addition, a MAC protocol utilizing priority-based power regulation [10], channel information-based back-off mechanism [11], and interference cancellation techniques [12] has been developed to mitigate interference in high-density wireless networks.

In this paper, we utilize a diversity combining scheme that uses wireless links between a station and non-associated APs to improve the performance of high-density wireless networks. Diversity combining techniques were proposed to improve the wireless transmission performance [2,3]. In [2], the authors proposed a block-based bit combining scheme in a multi-radio diversity (MRD) system consisting of multiple APs, clients, and an MRD combiner to combine frames from multiple APs at the MRD combiner. In block-based bit combining, each frame received by multiple APs is divided into blocks, and then the frame is reconstructed by assembling blocks until the reconstructed frame passes the CRC at the MRD combiner. However, because it has an exponential cost to correct errors as the length of a frame increases, the block-based bit combining scheme is not suitable for a practical system. In [3], the authors proposed a physical (PHY) layer symbol level combining technique to improve the reliability of transmission in wireless networks. They showed that their proposed scheme outperformed the state-of-the-art combining schemes in terms of BER. However, the PHY layer symbol level combining scheme requires more bits to represent physical layer information than bit combining schemes, which can cause significant overhead including additional transmission delay and processing delay for the diversity combining. Most studies focused only on the performance of diversity combining schemes and have not considered the MAC protocol perspective for feasibility.

## 3. System Model

**Figure 1:**
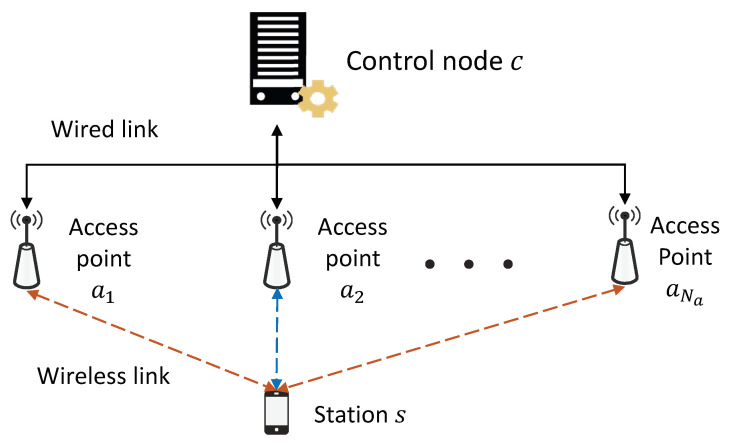
System model for a centralized wireless local area network with the control node.

We consider a centralized WLAN system that uses an infrastructure mode consisting of a CN and multiple APs and stations. The CN connects multiple APs over wired channels, and each AP provides wireless service to multiple stations. As shown in Figure 1, *c* denotes a CN; $a_{i}$ for $i\in \{1,2,\cdots ,N_{a}\}$ denotes an AP connected to the CN, where $N_{a}$ is the number of APs connected to the CN; and *s* denotes a station. In this system, we assume that all nodes in the WLANs follow the CSMA/CA protocol, and the stations are associated with the nearest AP. Furthermore, the APs and stations are arranged based on the homogeneous Poisson point process of  is the number of APs connected to the CN; and *s* denotes a station. In this system, we assume that all nodes in the WLANs follow the CSMA/CA protocol, and the stations are associated with the nearest AP. Furthermore, the APs and stations are arranged based on the homogeneous Poisson point process of $\Phi \subset R^{2}$ with intensity $\lambda_{a}$ and $\lambda_{s}$, respectively, in Euclidean planes.

In a saturated IEEE 802.11 DCF network, the channel access probability τ and conditional collision probability *p* are numerically given in [13]. In [13], τ and *p* are interdependent, and their relationship is given by $p=1-{\left(1-\tau \right)}^{n-1}$, where *n* is the number of stations in the WLAN. The channel access probability of all nodes is the same as τ because all nodes are assumed to be exposed to each other. When considering hidden stations, the conditional collision probability is given by $p=1-{\left(1-\tau \right)}^{c-1}{\left[{\left(1-\tau \right)}^{h}\right]}^{T_{s}}$, where *c* is the number of contending stations, *h* is the number of hidden stations, and $T_{s}$ is the approximate number of average slot decrements [14]. $T_{s}$ is the average time of successful transmission because the station attempts to transmit a data frame at the start of a time slot. The authors of [14] considered only networks with one access point and a limited number of stations in their numerical experiments; however, we consider a dense WLAN topology consisting of multiple APs in this paper.

In multiple HD-WLANs where multiple APs and stations are randomly distributed, when each station performs uplink data transmission, the station can have different numbers of contending and hidden nodes. This is because it is difficult to assume that all nodes are exposed to every other node in the multiple WLANs scenario. In addition, each contending node can be associated with different APs in the HD-WLANs. In other words, the conditional collision and channel access probabilities of each node can have different values because each node may have a different number of contending nodes in the transmission range and transmit a data frame to different APs. Let $n_{c}$ denote the number of contending nodes and $\tau_{i,c}\left(i=1,2,\ldots ,n_{c}\right)$ denote the channel access probability of the *i*-th contending node. Then, the probability that none of the contending nodes transmit a data frame is given by $\prod_{j=1,j\neq i}^{n_{c}}\left(1-\tau_{j,c}\right)$. On the other hand, the transmission duration of neighboring nodes of a hidden node can affect the channel access probability of the hidden node in HD-WLANs. If a node that is outside the carrier sensing range of an AP receiving the uplink transmission and is within the carrier sensing range of a hidden node transmits a frame, the hidden node does not access the channel, and thus the hidden node does not interfere with the reception of uplink data. Therefore, let $\alpha_{i}$ be the transmission duration of a neighboring node of a hidden node, the probability that a hidden node does not access the channel can be ${\left(1-\tau_{i,h}\right)}^{T_{s}-\alpha_{i}}$, where $\tau_{i,h}$ is the channel access probability of the hidden node, whereas the probability that all nodes affecting the hidden node do not access the channel is given by $P_{a}=\prod_{j=1}^{n_{a}}\left(1-\tau_{j,a}\right)$, where $n_{a}$ is the number of adjacent nodes affecting the hidden node and $\tau_{j,a}$ is the channel access probability of the adjacent node. Here, the expected value of the transmission duration is given by

(1) $$\bar{\alpha_{i}}=\left\{1-P_{a}\right\}\cdot \sum_{j=1}^{T_{s}}\left\{\left(T_{s}+1-j\right)\cdot {P_{a}}^{j-1}\right\}.$$

Consequently, the probability that none of the hidden stations access the channel is $\prod_{j=1}^{n_{h}}{\left(1-\tau_{j,h}\right)}^{T_{s}-\bar{\alpha_{j}}}$, where $n_{h}$ is the number of hidden nodes. Therefore, the conditional collision probability for multiple WLANs scenarios is given by

(2) $$p_{i}=1-\prod_{j=1,j\neq i}^{n_{c}}\left(1-\tau_{j,c}\right)\cdot \prod_{k=1}^{n_{h}}{\left(1-\tau_{k,h}\right)}^{T_{s}-\bar{\alpha_{k}}}.$$

Thus, the steady channel access probability can be obtained using (2).

In the HD-WLANs, when a station transmits a data frame to an associated AP, nodes outside the carrier sensing range of the station can be potential sources of interference. In particular, potential interfering nodes within the carrier sensing range of the associated AP can be critical because they interfere with data transmission at close distances. Let $\left\{t_{i}:i=1,2,\ldots ,N_{t}\right\}$ denote a set of nodes within the carrier sensing range of a station *s* that transmits a data frame after the contention and $\left\{r_{i}:i=1,2,\ldots ,N_{r}\right\}$ denote a set of nodes within the carrier sensing range of an AP associated with the station. When the station transmits a data frame, the set of potential interfering nodes can be represented as $\left\{u_{i}:i=1,2,\ldots ,N_{u}\right\}=\left\{r_{i}\right\}\backslash \left\{t_{i}\right\}$ using the difference between the two sets $\left\{r_{i}\right\}$ and $\left\{t_{i}\right\}$. To indicate whether the nodes of the previously defined interfering nodeset $\left\{u_{i}\right\}$ access the wireless channel when the station transmits a data frame, we define a channel access indicator set of the interfering nodes as $\left\{e_{i}:i=1,2,\ldots ,N_{u}\right\}$. If the element $e_{i}$ is equal to one, it means that the station accesses the wireless channel; if $e_{i}$ is equal to zero, the station does not.

Assuming that the interference signals from nodes outside the carrier sensing range of an AP associated with a station *s* are negligible, the received signals at the associated AP $a_{i}$ are defined using the channel access indicator set $\left\{e_{i}\right\}$ as follows:

(3) $$y_{i}=h_{s,a_{i}}\sqrt{P_{s}^{t}}d_{s}+\sum_{k=1}^{N_{u}}e_{k}\cdot h_{u_{k},a_{i}}\sqrt{P_{u_{k}}^{t}}d_{u_{k}}+N_{i},$$

where $d_{s}$ and $d_{u_{i}}$ are the transmission data of the station *s* and node $u_{i}$, respectively; $h_{s,a_{i}}$ and $h_{u_{k},a_{i}}$ are the channel coefficients between *s* and $a_{i}$ and between $u_{k}$ and $a_{i}$, respectively; $N_{i}$ is the additive white Gaussian noise with variance $\sigma^{2}$ at $a_{i}$; and $P_{s}^{t}$ and $P_{u_{i}}^{t}$ are the transmission power of the station *s* and interfering node $u_{i}$, respectively. If we assume that ${|h_{s,a_{i}}|}^{2}$ and ${|h_{u_{k},a_{i}}|}^{2}$ follow the exponential random distribution with mean value $g_{s,a_{i}}$ and $g_{u_{k},a_{i}}$, respectively, the SINR at the associated AP $a_{i}$ is obtained using (3) as

(4) $$\begin{array}{ccc} \Gamma_{a_{i}} & = & \frac{P_{s}^{t}\cdot {\left|h_{s,a_{i}}\right|}^{2}}{\sum_{k=1}^{N_{u}}e_{k}\cdot P_{u_{k}}^{t}\cdot {\left|h_{u_{k},a_{i}}\right|}^{2}+\sigma^{2}}. \end{array}$$

The average SINR can be obtained using the channel access probabilities obtained using Formula (2) instead of $e_{i}$. Further, the average BER of the M-ary quadrature amplitude modulation based on SINR values at $a_{k}$ is given in [15] by

(5) $$P_{b}^{k}≅\frac{\sqrt{M}-1}{\sqrt{M}\log_{2}\sqrt{M}}\mathrm{erfc}\left[\sqrt{\frac{3\log_{2}M\cdot \Gamma_{a_{k}}}{2(M-1)}}\right],$$

where erfc(·) is the complementary error function.

## 4. MAC Protocol for HD-WLAN

The following subsection describes the MAC protocol for applying simple link-layer diversity combining in HD-WLANs and an ACS mechanism to improve network throughput in the proposed system where diversity combining is applied.

### 4.1. MAC Protocol for Data Combining

**Figure 2:**
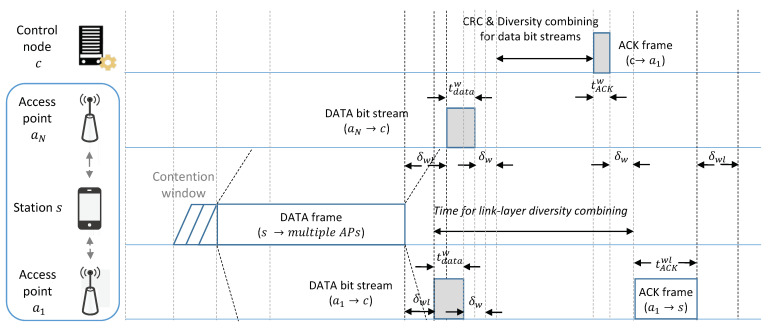
Medium access control (MAC) protocol using link-layer diversity combining for reliable data transmission.

We propose a MAC protocol in a centralized WLAN system to improve the reliability of data transmission in a HD-WLAN environment. The proposed protocol uses multiple APs close to each other for cooperative processing at a CN. In WLANs, protocols for the efficient use of frequency resources are required, because a large amount of interference can drastically degrade SINR and throughput performance if multiple APs receive data frames from different stations simultaneously with the same frequency band. The proposed MAC protocol can increase the reliability of data transmission by performing a diversity combining technique using the same data frame received from multiple APs through each wireless channel to improve spectral efficiency, unlike existing protocols that discard data transmission at unconnected stations. In order words, the CN performs error correction by using aggregated data from multiple APs in the proposed MAC protocol. Figure 2 shows the proposed data transmission procedure in the WLAN system when there are *N* APs close to each other; the white and gray boxes denote wireless and wired transmissions, respectively, and the AP $a_{1}$ and $a_{i}$ for i∈{2,⋯,N} denote the associated and non-associated APs connected to the CN, respectively. The procedure of the proposed protocol is as follows.

- The station *s* begins data transmission to the associated AP $a_{1}$ after the back-off process. The station transmits data to the associated AP; other APs within the transmission range of the station can also receive the data frame from the station.
- After receiving the data frame from the station, the AP $a_{1}$ performs a CRC. If the data frame passes the CRC, the AP sends an acknowledgment (ACK) frame to the station after a short interframe space (SIFS); otherwise, the AP forwards the data frame including the SINR information to the CN to correct the errors.
- The non-associated APs immediately forward the data frame including the SINR information to the CN after receiving the data frame from the station, even if the data frame does not pass the CRC and the destination address of the data frame does not match the address of the APs.
- After receiving the data frames from multiple APs, the CN performs error correction if the frame from the associated AP does not pass the CRC. In the error correction, the CN performs the CRC on all received data frames. If at least one of the received frames passes the CRC, the CN forwards the data frame to the upper layer and sends an ACK frame to the AP $a_{1}$; otherwise, the CN performs the diversity combining to correct errors using an SINR-based MVA. A detailed explanation of the combining scheme is given in Section 5.
- If the corrected bit stream passes the CRC, the CN sends the corrected data frame to the upper layer and an ACK frame to the AP $a_{1}$. Then, the AP $a_{1}$ receives the ACK frame from the CN, and the AP transmits it to the station. If the corrected bitstream does not pass through the CRC, the station *s* attempts to retransmit the data frame to the AP because it did not receive the ACK frame from the AP.

### 4.2. ACS Mechanism

In the CSMA/CA protocol, stations check the state of the channel through PCS to avoid frame collision with other stations and reduce the impact of interference from other stations. If the range of PCS is wide, the probability of frame collision decreases because the probability that the stations will transmit data, which determines that the channel is busy, increases. However, channel utilization owing to exposed terminal problems can be reduced, which can decrease network throughput. Thus, the problem of determining an appropriate PCS range that can reduce the collision probability and increase the utilization of the wireless channel is paramount for improving wireless network performance. To determine the appropriate PCS range that can improve network throughput in various WLAN environments, many studies on ACS mechanisms have been actively conducted [5].

**Figure 3:**
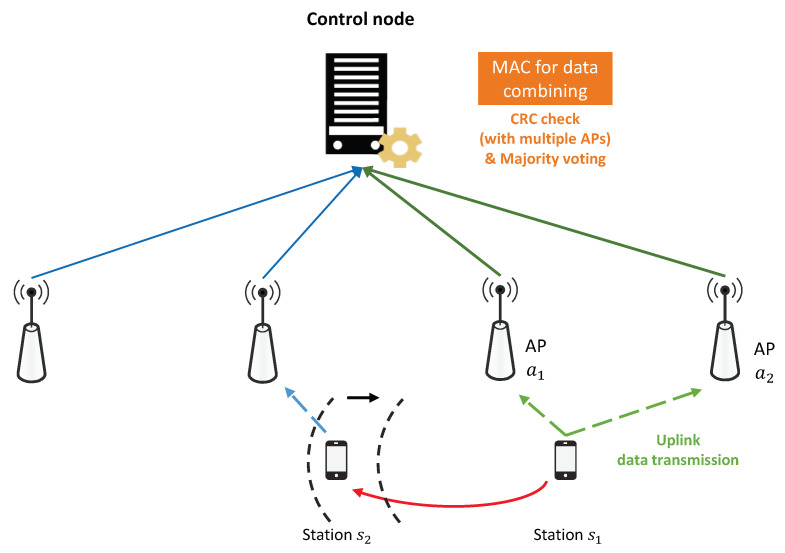
Carrier sensing range adjustment scenario in the WLAN system using the diversity combining scheme.

In the proposed system, the CN can modify the errors contained in the uplink data stream using the diversity combining scheme, even if the uplink data stream received from the station to the associated AP contains some errors by interference signals. In this case, as the associated AP is more robust against interference signals than the APs in the existing WLAN system, lowering the range of PCS to increase channel utilization can increase the gain in terms of uplink throughput performance. Figure 3 describes a scenario that mitigates the exposed terminal problem by adjusting carrier sensing. In the case of reducing the carrier sensing range of the WLAN nodes, uplink transmission of station $s_{1}$ can be successfully performed through the combining technique even if the influence of interference increases. In addition, as the station $s_{2}$ can perform uplink transmission at the same time with $s_{1}$, network throughput can be improved.

In the proposed system using the diversity combining scheme, as the CN continuously receives uplink data streams from the connected APs, it can calculate uplink throughput using the number of successfully received data streams over a period and their length. Therefore, we propose a MAC protocol for the ACS mechanism to obtain network throughput gains using uplink throughput calculated by the CN. Let $s_{i}$ be a binary value indicating successful reception of the *i*-th uplink data stream in the proposed system where the diversity combining scheme is applied. The equation for calculating the network throughput is as follows:

(6) $$UT=\frac{\sum_{i}^{N_{D}\left(t_{m}\right)}l_{i}\cdot s_{i}}{t_{m}},$$

where $l_{i}$ is the length of the *i*-th uplink data stream, $t_{m}$ is the measurement time for uplink throughput calculation, and $N_{D}$ is the number of uplink data transmissions performed in WLANs connected to the CN over the measurement time $t_{m}$. If $s_{i}$ is 1, it means that the *i*-th uplink data stream has been successfully received; if $s_{i}$ is 0, it means that the data stream reception has failed. The success rate of uplink transmission can be calculated by $R_{s}=\left(\sum s_{i}/N_{D}\left(t_{m}\right)\right)$. As the number of successfully received uplink data for a certain period increases, the uplink throughput increases. Therefore, as $N_{D}\left(t_{m}\right)$ and the success rate $R_{s}$ in (6) increase, the uplink throughput increases.

In the proposed ACS scheme, $N_{D}\left(t_{m}\right)$ and $R_{s}$ are increased by adjusting the carrier detection range.

In the proposed system, the WLAN nodes adjust the PCS threshold using the CN to adjust the PCS range. The procedure of MAC protocol for the proposed ACS adjustment scheme to improve the uplink throughput of (6) is as follows.

- The CN determines the minimum and maximum values of the PCS threshold, PCS threshold adjustment unit $P_{u}$, and measurement time $t_{m}$ for uplink throughput calculation. Then, the CN selects one or more initial PCS thresholds with equal intervals between the minimum and maximum values of the PCS threshold.
- The CN delivers the initial PCS threshold values to APs, the APs send the initial values to the stations, and then the WLAN nodes sequentially set the PCS threshold using the initial values. In the IEEE 802.11 standards, the APs broadcast the service set identifier value to nearby WLAN nodes. In this system, the PCS threshold value is included in the frames transmitted through the broadcast.
- The uplink throughput for each initial PCS threshold is calculated via (6) using the measurement time $t_{m}$ at the CN. After setting the PCS threshold value to derive the highest uplink throughput, the CN delivers the PCS threshold value to the APs, and the APs deliver the values to the stations.
- After $t_{m}$, the CN delivers the current PCS threshold minus $P_{u}$ to each AP and measures the uplink throughput again. If it is greater than the uplink throughput calculated in the previous iteration, the PCS threshold value decreases; if it is less than the uplink throughput calculated in the previous iteration, the PCS threshold increases. When the PCS threshold is increased using $P_{u}$, we obtain a reverse case.
- The process of adjusting the PCS threshold is repeated until convergence. After convergence, the PCS threshold is modified again after a certain time. The PCS threshold modification interval can be determined according to the network system conditions.

The proposed system using the diversity combining technique can improve the robustness against interference signals in dense WLANs. Thus, in the proposed protocol, the throughput gain of HD-WLANs can be further improved by adjusting the PCS range of the nodes in the WLANs according to the network environment.

## 5. AP Selection and Diversity Combining Algorithms

### 5.1. AP Selection Algorithm

We select an appropriate set of APs for diversity combining in HD-WLANs using the bit error probability. First, we derive the probability of success in the proposed bit-level diversity combining scheme based on MVA. The success probability depends on the set of APs participating in the diversity combining scheme among all APs connected to the CN. Therefore, once the set of APs participating in the diversity combining scheme is determined, the success probability of the diversity combining scheme can be derived for the AP set using the BER of the data frames received at each AP. Let **A** denote a set of APs as input for the diversity combining scheme and $x_{i}=\left[x_{i}^{1},x_{i}^{2},\cdots ,x_{i}^{N_{s}}\right]$ denote a binary vector indicating successful reception of the bit at APs within the transmission range of a station. Then, if more than half of the APs in the AP set **A** receive a data bit successfully, the output of the MVA yields a successful result. This can be expressed simply by formula as $\sum_{k=1}^{\left|A\right|}x_{i}^{k}>\frac{\left|A\right|}{2}$. The probability that $x_{i}^{k}$ is zero is equal to the bit error probability. In the proposed SINR-based MVA, if the number of APs is even and the summation of the elements is equal to $\frac{\left|A\right|}{2}$, the output is determined by the average SINR of the AP set. If a set with a higher average SINR has the correct bit, the proposed bit-level combining scheme can be used to obtain the correct bit. We define a set of possible binary vectors whose number of 1s is greater than or equal to $\frac{\left|A\right|}{2}$ as $X_{m}\left(A\right)$. Using (5) and $X_{m}\left(A\right)$, the success probability of the bit-level diversity combining is represented as

(7) $$P_{s}\left(A\right)=\left[\sum_{x\in X_{m}\left(A\right)}\prod_{k=1}^{\left|A\right|}{\left(1-P_{b}^{k}\right)}^{x^{k}}{P_{b}^{k}}^{1-x^{k}}\right],$$

where $x^{k}$ is the *k*-th element of the vector *x*. As shown in (7), the number of APs within the transmission range of the station can significantly influence the success probability of the bit-level diversity combining technology. A large number of APs used for diversity combining can increase the success probability of the scheme. However, if the CN uses the data stream of APs under severe channel conditions to perform the proposed bit-level diversity combining scheme, the performance of the proposed scheme can be drastically degraded. This is because the bit error probability of the data stream from the APs is high. Therefore, we propose an AP set decision algorithm for maximizing the success probability of bit-level diversity combining scheme as follows:

(8) $$\begin{array}{cccc} \; & \mathrm{maximize} & \; & P_{s}\left(A\right)\\ \; & \mathrm{subject}\;\mathrm{to} & \; & A\subset S, \end{array}$$

where **S** is the set of all APs connected to the CN within the transmission range of the station.

In the proposed AP set decision algorithm, the element of **A** is determined by sequentially containing the element of the AP with the lowest BER when calculating the success probability of the proposed bit-level diversity combining. Here, as the success probability $P_{s}\left(A\right)$ has a concave shape, (8) is a discrete optimization problem. Therefore, the point at which the value of $P_{s}\left(A\right)$ decreases can be selected as the optimal set. To solve the optimization problem of (8), Algorithm 1 describes the process of the proposed decision algorithm. First, for the diversity combining scheme, APs within the transmission range of the station are inputs. In lines 2–3, APs with a very low SINR values are filtered out for reliable the performance of diversity combining scheme. The SINR threshold for Algorithm 1 can be set depending on the system condition. The filtered APs are sorted in descending order based on the SINR in line 4, and we initialize the parameters before executing the loop in line 5. Then, a loop is run to find the point where the value of $P_{s}$ decreases. $P_{s}\left(\cdot \right)$ for this loop is calculated using (5). In lines 6–15, the AP with the lower BER value are included in the new AP set in order. If $P_{s}\left(A_{1}\right)$ is greater than $P_{s}\left(A_{2}\right)$, the set is determined as the optimal set for maximizing the performance of diversity combining. Therefore, the performance of bit-level diversity combining can be significantly improved even in severe channel conditions.
**Algorithm 1** AP set decision algorithm for maximizing the performance diversity combining1:$\mathrm{input}:\;\left[a_{1},a_{2},\cdots ,a_{N_{s}}\right]$2:filterAPswithavaluelowerthanthethresholdSINRasfollows:3:$\left[a_{1},a_{2},\cdots ,a_{N_{s}}\right]\to \left[a_{1},a_{2},\cdots ,a_{N_{s}^{*}}\right]$4:sortthefilteredAPsindescendingorderbasedontheSINRvalue5:$\mathrm{initialize}\;\mathrm{parameters};\;A_{1}\leftarrow \left[a_{1}\right],A_{2}\leftarrow \left[a_{1},a_{2}\right],i\leftarrow 2,\;\mathrm{and}\;\mathrm{calculate}\;P_{s}\left(A_{1}\right)$6:**while** $i\leq N_{s}^{*}$ **do**
7: $\mathrm{calculate}\;P_{s}\left(A_{2}\right)\;$
8: **if** $P_{s}\left(A_{1}\right)<P_{s}\left(A_{2}\right)$ **then**
9:  $A_{1}\leftarrow A_{2}$
10:  $A_{2}\leftarrow \left[a_{1},a_{2},\cdots ,a_{i+1}\right]$
11:  i←i+1
12: **else**
13:  $A^{*}\leftarrow A_{1}$
14: **end if**
15:**end while**16:$\mathrm{output}:\;A^{*}$

### 5.2. Diversity Combining Algorithm

We present a bit-level diversity combining scheme based on the MVA to correct uplink data streams due to interference from WLAN nodes. Let $b_{i}^{k}$ represent the *i*-th received bit at the AP $a_{k}$ and $N_{s}$ represent the number of APs within the transmission range of the station. The input bit vector for the MVA to correct the *i*-th bit is given by $b_{i}=\left[b_{i}^{1},b_{i}^{2},\cdots ,b_{i}^{N_{s}}\right]$. The MVA can be expressed as

(9) $$\mathrm{Majority}\left(\left[b_{i}^{1},b_{i}^{2},\cdots ,b_{i}^{N_{s}}\right]\right)=\left\lfloor \frac{1}{2}+\frac{\sum_{k=1}^{N_{s}}b_{i}^{k}}{N_{s}}\right\rfloor .$$

The output of MVA (9) becomes the corrected bit $b_{i}$. If the number of elements in the input vector is even and the number of elements with the value of 0 and the number of elements with the value of 1 is the same, it is difficult to obtain the output of MVA. To solve this problem, in the proposed SINR-based MVA, output is determined by the average SINR value for each group. In order words, if the average SINR of the group with element zero is higher than the average SINR of the group with element one, the output bit of the SINR-based MVA becomes zero; otherwise, it becomes one. Here, the computational complexity of the bit-level diversity combining based on the SINR-based MVA is represented as O(L) because the computation volume increases linearly as the length of input bitstream increases. To correct errors using the proposed MVA, at least half of the input bit vector $b_{i}$ must have the correct bits. The proposed diversity combining scheme can achieve high performance by performing the SINR-based MVA using frames delivered from the APs selected with the AP selection algorithm.

Further, the proposed diversity combining technique using MVA can be applied to wireless sensor networks. As the use of IoT technology increases, many wireless sensor nodes are being installed in a limited space, which causes severe performance degradation due to increased signal interference between sensor nodes [16]. To solve this performance degradation, the proposed diversity combining technique can be utilized to improve transmission reliability of sensor nodes. Moreover, most wireless sensor network protocols are designed to transmit short signals in a short time because wireless sensor network protocols place importance on power saving. Therefore, the proposed bit-level diversity combining scheme is suitable for wireless sensor networks because the proposed scheme can transmit a relatively short signal relative to symbol-level diversity combining schemes.

## 6. Overhead Analysis

We perform an overhead analysis to verify compatibility with existing MAC protocols. For compatibility with legacy WLAN nodes, stations should receive an ACK frame within the ACK timeout, even if the error correction is performed. In the proposed MAC protocol, the overhead time for the error correction is given by

(10) $$T_{o}=2\cdot \delta_{w}+t_{\mathrm{data}}^{w}+t_{\mathrm{ACK}}^{w}+H,$$

where $\delta_{w}$ is propagation delays at the wired link between the associated AP and CN; $t_{\mathrm{data}}^{w}$ and $t_{\mathrm{ACK}}^{w}$ are the transmission delays for data and ACK frames over the wired link, respectively; and *H* is the processing time of the error correction scheme including the CRC and diversity combining based on MVA. The station should receive an ACK frame within the ACK timeout to ensure a successful data transmission. Notably, ACK timeout is given by $2\cdot \delta_{\max}+t_{\mathrm{ACK}}^{wl}+\mathrm{SIFS}$, where $\delta_{\max}$ is the maximum propagation delay in the WLAN, and $t_{\mathrm{ACK}}^{wl}$ is the transmission delay for the ACK frame over the wireless link. Then, the processing time for error correction has to satisfy the following condition:

(11) $$H\leq \mathrm{SIFS}+2\cdot \left(\delta_{\max}-\delta_{w}-\delta_{wl}\right)-t_{\mathrm{data}}^{w}-t_{\mathrm{ACK}}^{w},$$

where $\delta_{wl}$ is the propagation delay at the wireless link between the AP and station. As shown in Figure 2, the propagation delay of the wireless link can vary depending on the location of the APs. In (11), $\delta_{wl}$ becomes the largest propagation delay time among wireless links between the station and APs receiving the data frame. For instance, suppose that the distance of the wired link between AP and CN is 500 m, the length of a data frame is 1440 bytes, and the length of an ACK frame is 14 bytes. Then, $\delta_{w}$ is calculated as 2.5 µs, and $t_{\mathrm{data}}^{w}$ and $t_{\mathrm{ACK}}^{w}$ are 1.12 µs and 0.01 µs, respectively, in a 10 gigabit Ethernet link. The right side of inequality in (11) is greater than 3 µs, even if $\delta_{wl}$ is equal to $\delta_{\max}$ because the SIFS is set to 10 µs at 2.4 GHz frequency band in IEEE 802.11n standard. This shows that WLAN nodes using the proposed MAC protocol can work with conventional WLAN nodes if the processing time is shorter than 3 µs.

In addition, implementing symbol-level diversity combining using the wireless channel information contained in symbols can achieve low BER. However, because symbols contain wireless channel information, the overhead is greater than expressing the same amount of information in bits. The symbol represents a two-dimensional signal, which has an in-phase and quadrature (I-Q) value. The AP needs 32 bits to send a single raw symbol expressed as an I-Q value to the CN over the wired link [3]. Therefore, as the length of the data frame to be transmitted increases, satisfying (11) becomes difficult owing to the increasing value of $t_{\mathrm{data}}^{w}$. For example, in the case of 16-quadrature amplitude modulation (QAM), because one symbol represents 4 data bits, the overhead is 8 times larger when forwarding information representing symbols than forwarding information representing data bits. In other words, for the 16-QAM, a packet of 11,520 bytes must be transmitted to deliver a data payload of 1440 bytes, which requires 8.96 µs for the transmission delay instead of 1.12 µs in a 10 gigabit Ethernet link. As a result, $t_{\mathrm{data}}^{w}$ increases, making it difficult to satisfy (11).

**Figure 4:**
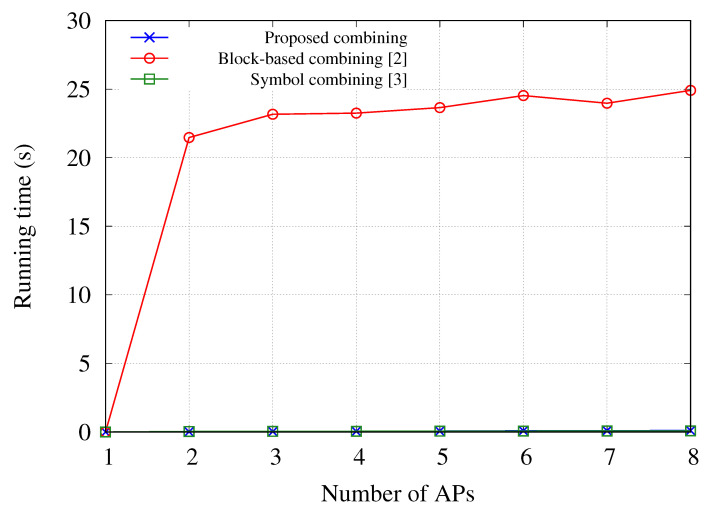
Running time on the CN to execute diversity combining schemes with respect to the number of APs [2,3].

Figure 4 shows the running time of the block-based bit-level diversity combining in [2], the symbol-level diversity combining in [3], and the proposed bit-level diversity combining. In this simulation, we developed the diversity combining schemes using MATLAB and measured the running time on a workstation (3.6 GHz quad-core processor and 8 GB RAM memory). The nodes use an orthogonal frequency-division multiplexing (OFDM) physical layer along with 16-QAM for wireless transmission in the diversity combining schemes, and the length of data frame is 1440 bytes. In addition, the average SINR at APs is set to 7 dB. The number of blocks used in [2] is set to 16, and the symbol-level diversity combining uses the centroid algorithm [3]. As shown in Figure 4, the running time of the diversity scheme proposed in [3] and the proposed diversity scheme has very low values, while the running time of the block-based diversity combining scheme proposed in [2] has relatively very large values. This is because the computational cost of reassembling a frame in block units until a frame passing the CRC is found is much higher than that of the other diversity combining schemes. The running time of the proposed scheme was slightly smaller than that of the symbol-level combining, and the difference between the symbol-level combining and the proposed combining schemes was within 0.01 s.

**Figure 5:**
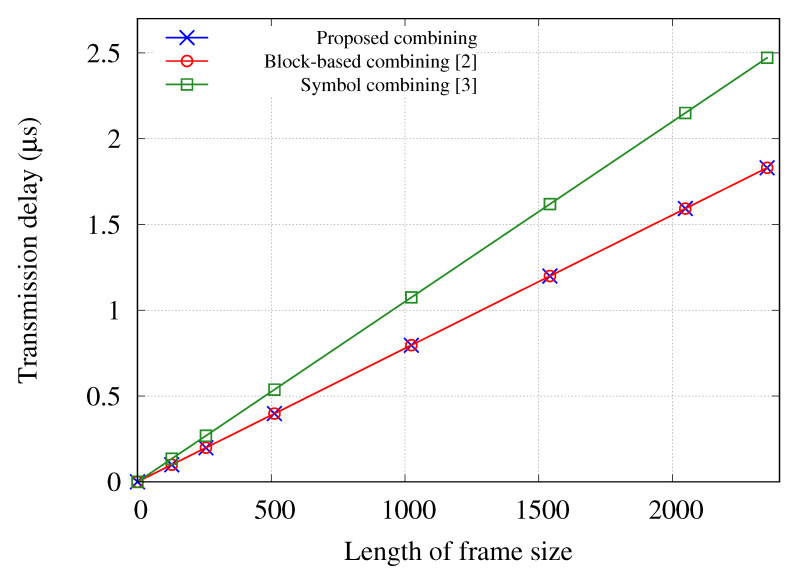
Transmission delay to forward data frames for diversity combining with respect to the length of the frame [2,3].

Figure 5 shows the transmission delay with respect to the payload length for different diversity combining schemes in a 10 gigabit Ethernet link. As shown in the results of Figure 5, the transmission delay of block-based and the proposed combining schemes is the same because both schemes are based on bit-level combining. On the other hand, although the low-fidelity symbol representation technique proposed in [3] significantly reduces overhead than conventional symbol-level combining schemes, the transmission delay of the symbol-level combing scheme is still more than 35% larger compared to the bit-level combing schemes. This is because the symbol-level combining scheme requires additional bits to represent symbol data. For compatibility with the existing WLAN nodes, the processing time and transmission delay should be minimized. Therefore, because the proposed scheme has low computational complexity and performs the MVA-based bit-level combining, it is the most suitable diversity combining for compatibility with conventional WLAN nodes.

## 7. Performance Evaluation

### 7.1. Simulation

We conducted simulation studies of the proposed error correction and ACS schemes using MATLAB. In these simulation studies, the minimum and maximum contention window sizes are set to 64 and 1024, respectively. The path loss exponent is set to 4. The transmission and noise power are −20 and −80 dBm, respectively. The grid size is 10 × 10 km, and the carrier sensing range is set to 100 m in the simulation environment. The threshold SINR for successful data transmission is set to 15 dB.

**Figure 6:**
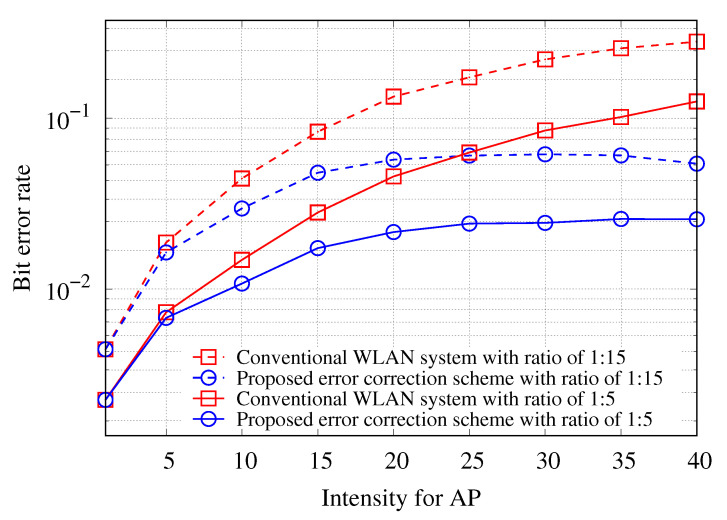
BER of uplink transmission with respect to intensity of AP at the ratio of the different the AP intensity and the station intensity.

Figure 6 shows the average BER of uplink transmissions with respect to the intensity of APs $\lambda_{a}$ to verify the performance of the proposed error correction scheme using link-layer diversity combining. In the simulation environment, as the intensity of APs $\lambda_{a}$ increases, the intensity of the stations $\lambda_{s}$ also increases at constant ratios of 1:5 and 1:15. The simulation results show that the average BER of uplink transmissions for the ratios 1:5 and 1:15 increases with $\lambda_{a}$, if the proposed error correction is not applied. This is because as the density of the nodes increases, the number of neighboring nodes using the same frequency band also increases, resulting in an increased interference level. Moreover, the proposed error correction scheme yields a considerably lower average BER than the other schemes. The difference in the average BER between without and with the error correction scheme increases with increasing intensity $\lambda_{a}$. This is because the proposed error correction algorithm using the link-layer diversity combining technique is performed on the CN of the centralized WLAN to correct errors caused by interference. Thus, as shown in Figure 6, the proposed error correction scheme drastically lowers the average BER of uplink transmission in HD-WLANs.

**Figure 7:**
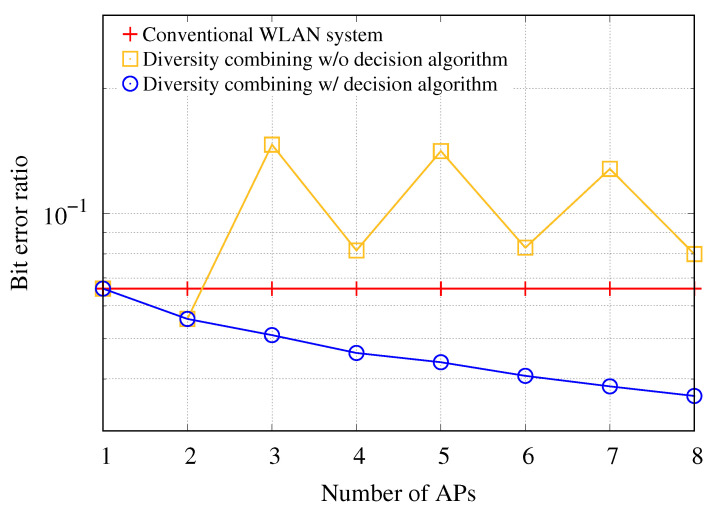
BER of uplink transmission with respect to number of APs participating in diversity combining for different combining policies.

To verify the performance of the AP set decision algorithm for a bit-level diversity combining scheme, we compare the combining scheme with and without the decision algorithm in terms of BER. In this simulation, the average SINR for the associated AP with the transmission station is set to 5 dB, and the average SINR for other APs within the transmission range of the station is set to 0 dB. Figure 7 shows the BERs of uplink transmission for the AP in conventional WLAN and the APs with the diversity combining scheme with and without the decision algorithm. If the diversity combining scheme is not applied, the BER is constant regardless of the number of APs. Meanwhile, if the diversity combining scheme without the decision algorithm is used in the WLANs, the results show the highest BER in cases where the number of APs is greater than 2. Under unstable channel conditions, frames with low SINR are more likely to contain many error bits, leading to poor performance of the diversity combining scheme even if the CN uses the information of a larger number of APs when performing the diversity combining scheme. If the diversity combining scheme using the decision algorithm is used for error correction, the results show the smallest BER because the proposed decision algorithm helps to perform the diversity combining scheme using only bitstreams that can improve the performance of the diversity combining among bitstreams delivered from the APs.

**Figure 8:**
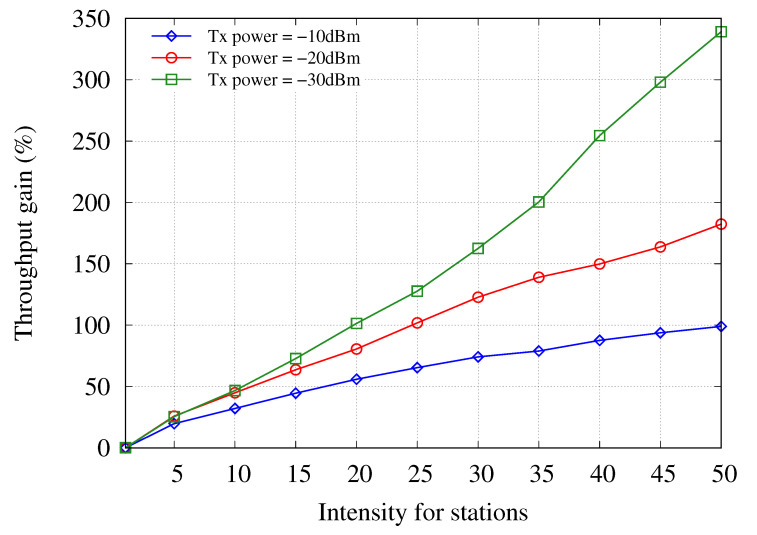
Throughput gain of the proposed adaptive carrier sensing scheme with respect to the intensity of stations for different transmit powers.

Figure 8 shows the uplink throughput gain of the proposed ACS scheme according to the intensity of the stations when the transmission power is set to −10, −20, and −30 dBm. The throughput gains were calculated using the increased uplink throughput in the network environment, where the proposed ACS technique was introduced and compared with the uplink throughput in the network environment where the carrier sensing range is fixed at 100 m. In this simulation environment, the intensity of the AP is set to 40, and the intensity of the stations on the x-axis is the intensity of the stations per AP. As shown in Figure 8, the throughput of the proposed ACS scheme was improved in all cases. In the proposed system, as the robustness to interference is improved by the diversity combining scheme, the number of exposed nodes can be reduced by making the carrier sensing range smaller. Thus, the proposed ACS scheme can improve throughput by increasing the number of uplink transmissions that simultaneously succeed through adjustment of the carrier sensing range. Furthermore, as the transmit power decreases, the throughput gain increases because the number of uplink transmissions that succeed at the same time increases.

### 7.2. Experiment

**Figure 9:**
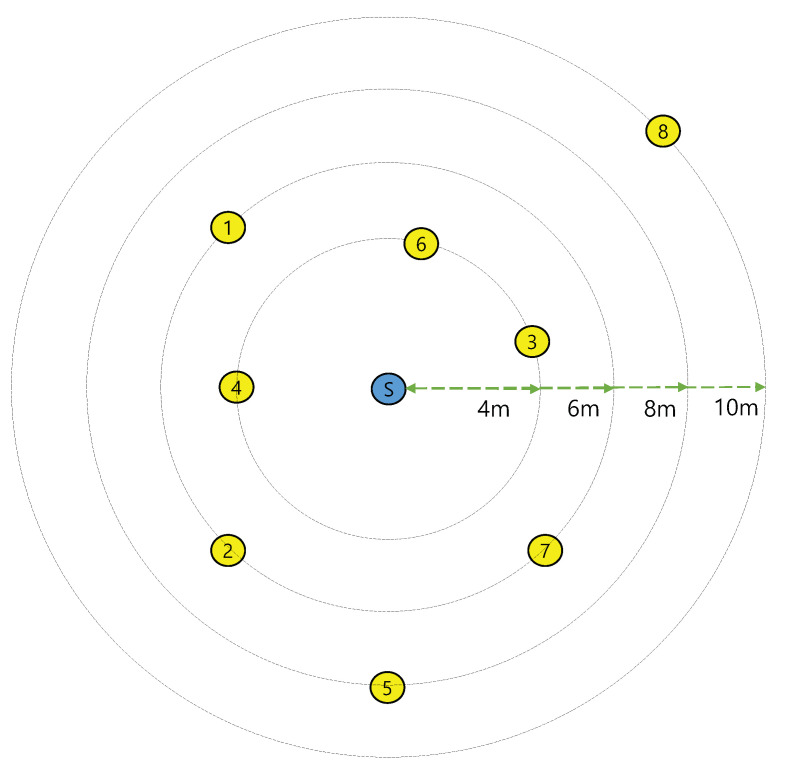
Network topology for configuring the experimental environment using WARP hardware devices.

**Figure 10:**
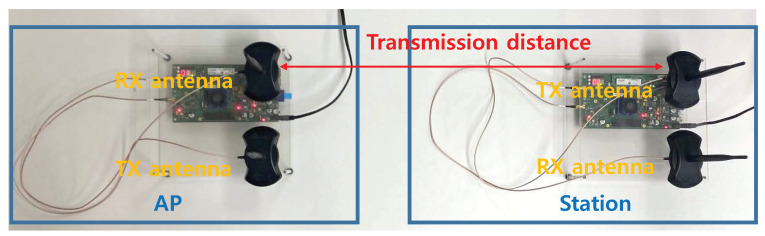
SDR-based experiment using WARP v3 board.

Experiments using SDR were conducted based on the wireless open-access research Platform (WARP) to validate the performance of the proposed link-layer diversity combining scheme [17]. We implemented the experimental environment using the WARPLab framework with WARP v3 devices; all devices use a carrier frequency of 2.4 GHz with the OFDM physical layer and a quadrature phase shift keying modulation scheme. The data stream used for this experiment is 1440 bytes long, and the number of APs within the transmission range was set to 8. As shown in the network topology in Figure 9, the APs were placed and the station was placed at the center. The distances between the station and APs are 4, 6, 8, and 10 m, respectively. The station and APs are implemented using WARP v3 hardware boards pictured in Figure 10. The APs receive the data frames from the station, and then forward the frames through Ethernet to a workstation acting as the CN. The workstation performs the proposed diversity combining using the data frames from multiple APs.

**Figure 11:**
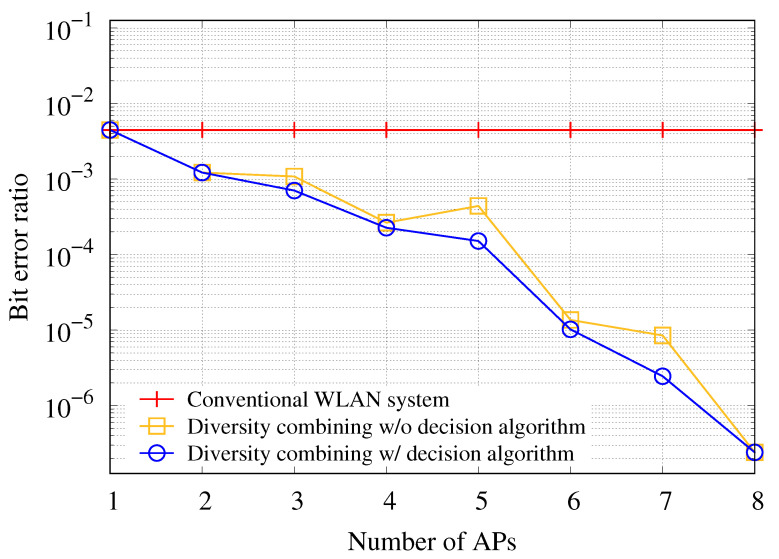
BER of uplink transmission with respect to number of APs for the different diversity combining schemes in the testbed using the WARP devices.

Figure 11 shows the BER obtained using the SDR-based testbed in WLANs to experimentally verify the performance of the proposed diversity combining. When the diversity combining scheme is not used, the BER of the associated AP remains constant regardless of the number of APs. On the other hand, in all cases where the number of APs is 2 or more, when the proposed diversity combining scheme is used, the BER becomes much smaller than the BER obtained when the diversity combining is not used. This is because more information is used to correct the error bits as the number of APs increases. As shown in Figure 11, the diversity combining scheme using the AP set decision algorithm has a lower BER than the diversity combining scheme using data frames from all APs. Using data frames forwarded from all APs to perform diversity combining can degrade the combining performance if data frames containing a large number of errors received through unstable wireless channels between APs and the station. On the other hand, the proposed decision algorithm can filter out received data frames through the unstable wireless channels, showing the lower BER.

## 8. Conclusions

This paper has proposed the MAC protocol that utilizes a link-layer diversity combining scheme in the centralized WLAN system consisting of the CN and multiple APs to improve the performance of HD-WLANs. For compatibility with conventional MAC protocols, we have designed the scheme to minimize the overhead for diversity combining, and it showed lower overhead than conventional combining schemes through simulation studies. We have also proposed the ACS mechanism to improve uplink throughput in WLAN system where diversity combining is used. The simulation results showed that the performance of the proposed ACS mechanism is significantly improved in high-density wireless networks. To experimentally verify the performance of the proposed link-layer diversity combining scheme, we have conducted experiments using SDR equipment, and the experimental results showed an improvement in reliability of wireless transmission.
